# The effect of the sense of Community on Depression, Anxiety, and Stress: the mediation role of a balanced time perspective

**DOI:** 10.1186/s40359-024-01797-4

**Published:** 2024-06-01

**Authors:** Luigi Micillo, Natale Canale, Davide Naddeo, Nicola Cellini, Giovanna Mioni

**Affiliations:** 1 Department of General Psychology, University of Padova, Via Venezia 8, Padova, 35131 Italy; 2 Department of Developmental and Social Psychology, University of Padova, Padova, Italy

**Keywords:** Balanced time perspective, Time perspective, Sense of community, Depression, Anxiety, Stress

## Abstract

**Purpose:**

Social context and time are two dimensions within which our entire existence is embedded. Therefore, prompting a positive set of attitudes and beliefs towards these elements is fundamental for individuals’ psychological well-being. Currently, there is limited understanding regarding the interplay between the sense of community and time perspective in relation to psychological distress. The present study aims, at investigating the effects that the sense of community and time perspective have on the levels of anxiety, depression, and stress. Particular attention has been dedicated to testing whether the effect of sense of community on anxiety, depression, and stress is mediated by the deviation from a balanced time perspective.

**Methods:**

To accomplish our purposes, we asked 352 participants to complete an online survey and respond to the Depression, Anxiety, Stress Scale (DASS-21), the Zimbardo Time Perspective Inventory (ZTPI), and the Multi-Dimensional Sense of Community Scale (MTSOCS). From these scales, we obtained the scores for anxiety, depression, and stress as well as a general score for the sense of community and the deviation from a balanced time perspective. We computed three General Linear Mediation Models, one for each scale of the DASS-21.

**Results:**

The results showed that the relationship between sense of community and psychological distress was mediated by the deviation from a balanced time perspective extending previous findings and enriching the existing literature on time perspective.

**Conclusion:**

The results described so far could be applied to build a series of interventions aimed at promoting psychological well-being in the general population. Considering our findings, we suggest that individuals’ health could be promoted by both improving their sense of community, which in turn would decrease their levels of stress, and by restructuring their time perspective when it became dysfunctional and unbalanced.

## Introduction

In contemporary society, mental health has emerged as a critical concern as the prevalence of psychological disorders continues to rise [1, 2]. High levels of anxiety, depression, and stress impose significant limitations on individuals’ well-being, hindering their ability to experience a fulfilling life and pursue their goals [3]. Experiencing psychological distress can significantly diminish one’s quality of life, leading to adverse effects on interpersonal relationships, overall personal satisfaction, emotional well-being, and occupational performance [4]. Moreover, it can also result in decreased cognitive abilities and coping strategies [5]. The rise in the levels of anxiety, depression, and stress can be attributed to different factors. Besides some temperamental factors (e.g., negative affectivity) as well as environmental factors (e.g., adverse childhood experiences) [6], some authors have also pointed out the possible contribution of subjective Time Perspective [7] and Sense of Community [8] on psychological distress. Our actions and decisions are deeply intertwined with the concept of time. Our decision-making processes are influenced by past experiences, and we contemplate the potential consequences of our choices on our future. Consequently, the attitudes we adopt toward the past, present, and future play a crucial role in shaping our psychological functioning and influencing how we respond to external and social stimuli [9, 10]. Furthermore, being social creatures, humans are naturally inclined to engage in interpersonal relationships with others at different levels, ranging from our close-knit neighbourhoods to entire cities and even beyond [11]). Besides the fundamental relevance of social context and attitude toward time in our everyday lives, little is known about the relationship between either the sense of community and time perception alone or their connection with psychological well-being. The purpose of the current work is, thus, to evaluate the association of Time Perspective and Sense of Community with the levels of anxiety, depression, and stress.

### Time perspective

When speaking of Time Perspective, we often refer to the theory developed by Philip Zimbardo and John Boyd that considers Time Perspective as “*the often-nonconscious process whereby the continual flows of personal and social experiences are assigned to temporal categories, or time frames, that help to give order, coherence, and meaning to those events*” [7, p.1271]. The time frames highlighted by Zimbardo and Boyd are five, each one reflecting a specific set of attitudes toward the past, present, and future. The five time-frames are: on the one hand *Past Negative* (PN) reflects a regretful view of past experiences, and on the other hand *Past Positive* (PP) reflects a positive and nostalgic attitude towards the past. Furthermore, the authors describe the *Future* (F) time frame that reflects a general concern for the future, whilst the fourth category described by the authors is the *Present Hedonistic* (PH) which describes individuals who are more oriented to the enjoyment of the current moment, seeking for deeper sensations. The last category identified is the *Present Fatalistic* (PF) which reflects a hopelessness, deterministic, and catastrophic attitude towards the present. The five sets of attitudes regarding temporal horizons have been discovered to be significant predictors of psychological well-being. In the original validation of the instrument, Zimbardo & Boyd [7] found that higher scores on the PF and PN were both moderately linked with anxiety and depression. Desmyter & De Raedt [12] investigated the association of time perspective with psychological well-being and found that a negative set of attitudes towards the past was positively associated with negative affect and depression. Moreover, a negative attitude towards the past was negatively correlated with satisfaction with life. In the same study, the authors observed also that a more fatalistic attitude towards the present was positively related to the reported levels of the sample [12]. Papastamatelou and colleagues [13] showed that participants diagnosed with generalized anxiety disorder reported higher scores on the F subscale in comparison to those who did not receive a psychiatric diagnosis. Moreover, the authors showed a positive correlation between PN and PF and the reported levels of stress [13]. Similarly, in a study conducted by Micillo and colleagues [14], it was found that PN, PF, and F, positively predicted the reported levels of anxiety. Moreover, PN, PF, and PP predicted the reported values of depression as well [14]. Zimbardo and Boyd [7] also introduced the concept of Balanced Time Perspective (BTP), which refers to a harmonious and adaptive approach individuals adopt in considering their past, present, and future experiences. It involves maintaining a healthy integration of positive elements from each temporal dimension, avoiding excessive focus on any one aspect, and making decisions that lead to overall well-being and fulfilment.

### Sense of community

The psychological Sense Of Community (SOC) is seen as another important factor impacting psychological distress. The concept of the SOC has to be intended as a multidimensional construct that describes the “*feeling that members have of belonging, a feeling that members matter to one another and to the group, and a shared faith that members’ needs will be met through their commitment to be together*” [8, p.9]. The conceptualization of the SOC given by McMillan and Chavis [8] comprises four elements: the sense of belonging and sharing a sense of relatedness that the authors define as *Membership*, the reciprocal sense of mattering between a group and its members that is defined as *Influence*, the sense that the needs of the individuals will be pursued by the group that is defined as *Integration and Fulfilment of needs*, and, finally, the sense of commitment and belief that the members of the group have towards each other that is defined by the authors as *Shared Emotional Connection* [8]. Previous studies found that a strong SOC fosters positive mental health in adults [15] regardless of the negative events they experienced [16]. For example, among the four dimensions it has been found in a community sample that those individuals who felt to belong more to their community reported decreased levels of depression and that depressive symptoms lasted less longer [17]. In addition, a study on a sample of older earthquake survivors in China reported the moderation effect of SOC between a traumatic experience (earthquake-associated distress) and depressive symptoms [18]. SOC has also an effect on stress levels: both Garcia-Cid et al. [19] and Benson & Withson [20] found that higher levels of SOC are negatively associated with the level of stress, respectively in a sample of immigrants and a sample of college students [19, 20]. Previous research has also investigated the relationship between SOC and anxiety: a longitudinal study on a sample of heterosexual couples found that, in the context of the COVID-19 pandemic, SOC levels during the first wave are associated with lower levels of anxiety in the second wave for both partners. For female partners, this effect extends to the third wave [21]. The studies presented so far highlight the negative relationships between SOC and depression, anxiety, and stress. It is nonetheless important to note that most of the studies refer to specific groups (e.g. disaster survivors and immigrants), a condition that makes the results difficult to be extended to the general population.

### Aims and hypotheses

The relevance of time perspective and the set of beliefs we have of towards community in our lives is undoubted. All our actions are indeed embedded within those two dimensions, we rely on temporal information to perform even the simplest action, and we act in a social environment that influences and is influenced by us. Therefore, a good ability to orient and live in these two dimensions is essential for individuals’ physical and psychological well-being. However, previous studies have investigated the involvement of time perspective and sense of community on anxiety, depression, and level of stress independently without studying the possible association of these two relevant dimensions with psychological well-being. The psychosocial mechanisms by which sense of community and time perspective might influence psychological distress are not clearly understood. Thus, the present study aimed to explain the potential effects of environmental factors (e.g., SOC) on psychological distress by testing possible associations with time perspective. Undoubtedly, the present study can serve as a starting point for research in positive psychology, aiming to understand the intricate relationship between the feelings and beliefs we hold towards our community, as well as temporal horizons. As far as we are aware, to date, this knowledge is lacking. Therefore, we designed the present study to investigate the effects of the SOC and BTP on the levels of anxiety, depression, and stress. Further, we want to investigate whether the effect of SOC on anxiety, depression, and stress is mediated by the deviation from a BTP.

We hypothesize that individuals who experience a poorer SOC are more likely to report higher levels of anxiety, depression, and stress [17]. We expect that levels of time perspective that deviate more from optimal ones may be associated with higher levels of anxiety, depression, and stress. Put together, we also hypothesize that the role that time perspective has on psychological distress mediates the effect that sense of community has on anxiety, depression, and stress.

## Methods

### Participants

With the aid of social media pages and a snowballing sampling technique, we obtained a sample of 352 participants (230 females) who completed our online survey administered through Google Forms. The age of the participants ranged from 18 to 86 years old with a mean of 35.51 (SD = 15.43) years. All participants gave their online consent before starting the survey. Furthermore, the study was developed in accordance with the Declaration of Helsinki and was approved by the Ethical Committee of the University of Padova (n 3802).

### Measures

After consenting to participate, and providing some sociodemographic information, participants were asked to complete three questionnaires: the 21-item version of the Depression, Anxiety, and Stress Scale (DASS-21 [22, 23]), the Zimbardo Time Perspective Inventory (ZTPI [7, 24]) and the Multi-Dimensional Sense of Community Scale (MTSOCS [25]).

### Depression, Anxiety, Stress Scale - DASS21

The Depression, Anxiety, Stress Scale was developed by Lovibond & Lovibond [22] for differentiating anxiety, stress, and depression symptoms. The scale we employed in the present study is the reduced version composed of 21 items (DASS-21) divided into three subscales: anxiety, depression, and stress. The 21 items required participants to respond along a four-point Likert scale to how much the events happened in the previous week ranging from 0 (it never happened to me) to 3 (it always happens to me). The translation used for this study was the one developed by Bottesi and colleagues (2015) [23]. The reliability values of the scale ranged from *α* = 0.73 to *α* = 0.90 [23].

### Zimbardo time Perspective Inventory - ZTPI

The Zimbardo Time Perspective Inventory (ZTPI) is a scale developed by Zimbardo and colleagues in 1999 composed of 56 items that subtend five subscales – Past Positive, Past Negative, Present Fatalistic, Present Hedonistic, and Future – describing each a specific attitude towards Past, Present, or Future. Each item required participants to respond along a five-point Likert scale ranging from 1 (very untrue) to 5 (very true). In the present study, the Italian translation [24] was employed and the BTP was computed according to the model proposed by Jankowski and colleagues [26] obtaining the Deviation from a BTP (DBTP) with the following formula:

$$\sqrt{{(oPN - ePN)}^{2} + {(oPP - ePP)}^{2} + {(oPF - ePF)}^{2}+ {(oPH - ePH)}^{2} + {(oF - eF)}^{2}}$$

The formula presented above computes the DBTP as the Euclidean distance between optimal (*o*) and observed (*e*) levels of time perspectives [26]. In the present work, the optimal levels are the ones described by Jankowski et al. [26] having the following optimal values: PN = 1, PP = 5, PF = 1, PH = 3.4, and F = 5. Cronbach’s *α* has been computed for all the subscales and the values obtained ranged from *α* = 0.65 to *α* = 0.84.

### Data analyses

The hypotheses were tested using General Linear Mediation Models which were implemented in the R environment (https://www.r-project.org/) employing functions imported from *stats* [27], *mediation* [28], and *car* [29] packages. At first, we computed a multivariate skewness and kurtosis analysis by performing a Mardia Test on the variables of interest. Since not all the variables were properly distributed, we decided to use on the non-normal variables a Generalized Linear Model Approach. Then, a correlation analysis was performed in order to understand the relationship among the variables.

For each dependent variable, a mediation model has been computed as follows: first, the total effect has been computed with a glm function for Gamma family. Then, a lm function has been employed to compute the effect of the Independent Variable on the mediator. Finally, we tested the effect of the mediator on the dependent variable, keeping constant the Independent Variable again with a glm function for Gamma distributed data. The Causal Mediation analysis has been computed using the mediate function of the mediation package in R. Further, to avoid multicollinearity in multiple regression models we computed the Variance Inflation Factors (VIF) for the variables before running the mediation models. We considered that multicollinearity was not a problem in our model when VIF *<* 2.5 [30].

## Results

**Fig. 1:**
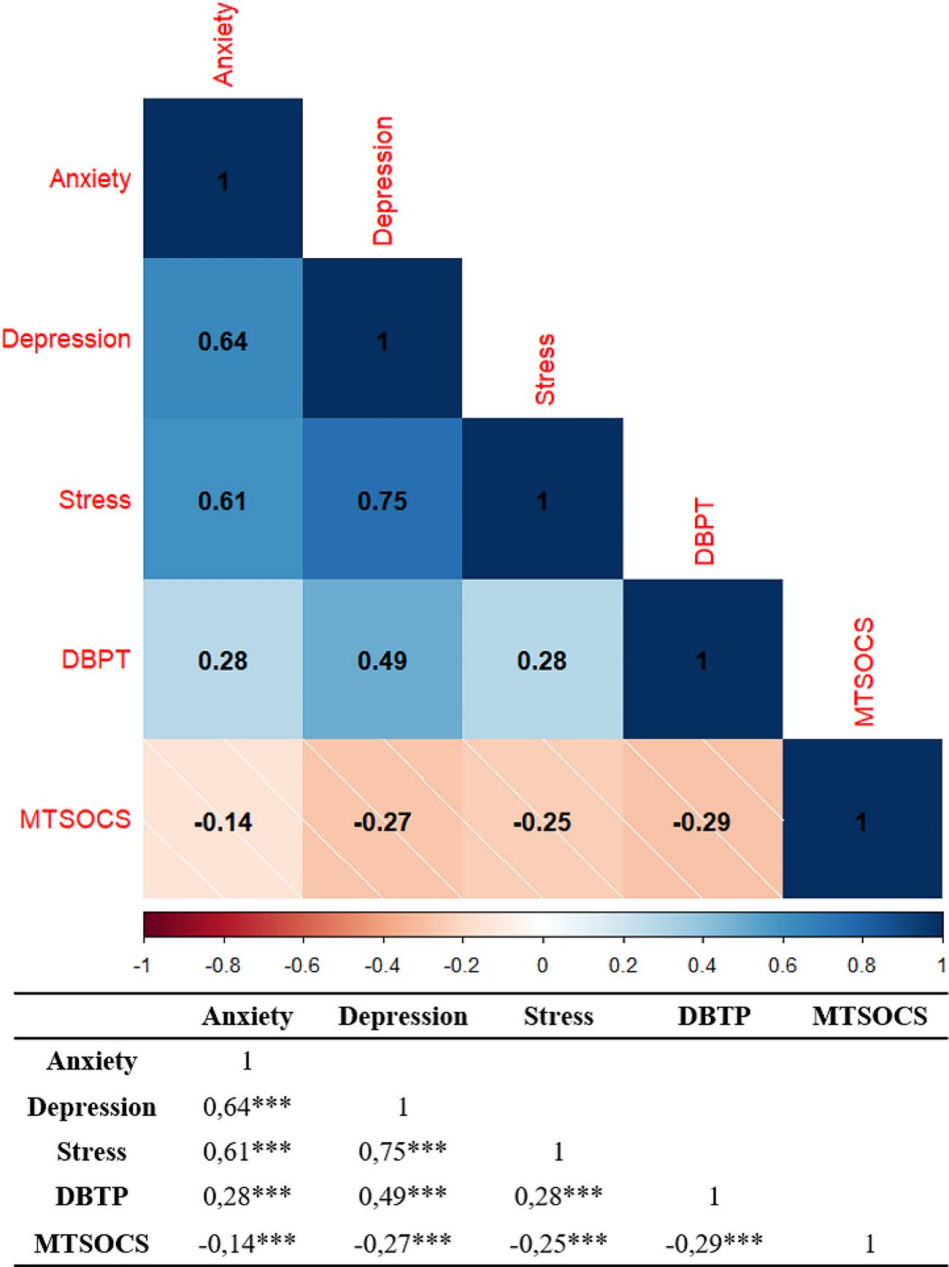
Correlation Matrix of the variables included in our models. DBTP: Deviation from Balanced Time Perspective; MTSOCS: Multidimensional Sense of Community Scale. *Note* *** indicates p values < 0.001

First, to understand the relationship among the variables, we computed a correlation matrix to explore whether or not the variables included in our model were related. As shown in Fig. 1, we observed that there was a negative relation between the total score of the SOC and the other variables, whilst higher values in the DBTP were associated positively with anxiety, depression, and stress.

To understand the possible relation between the SOC and anxiety, depression, and stress, and for further investigating the mediation role of DBTP we ran a mediation model for each of the three dimensions of the DASS-21 having the total score of the MTSOCS as independent variable and the DBTP as mediator.

### Anxiety

**Fig. 2:**
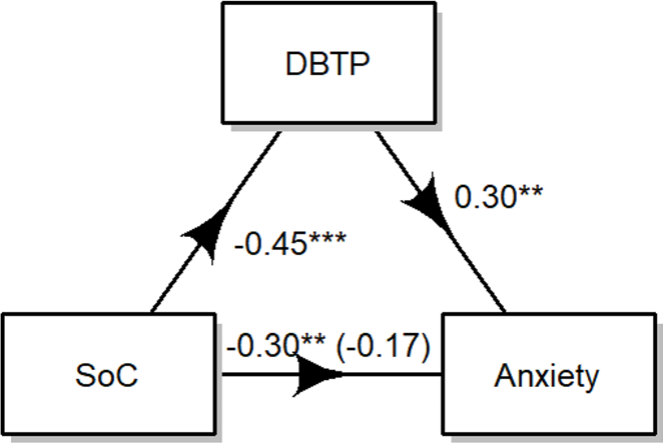
Mediation Model of DBTP on the effect of SoC on anxiety. *Note* *** indicates p values < 0.001; ** indicates p values < 0.01

The results showed that the impact of SoC on anxiety is mediated by the DBTP. As can be seen in Fig. 2, the regression coefficient between DBTP and anxiety (*B* = 0.30, β = 0.46, t (351) = 4.84, D = 21.39, R^2^ = 0.02, *p <* .01), was found to be significant. No significant effect was found concerning the regression coefficient of SoC and anxiety (B=-0.18, β = -0.27, t (351) = -1.79, D = 21.39, R^2^ = 0.02 *p* = .08) The estimated indirect effect was − 2.39. The significance of this effect was assessed using bootstrapping procedures. Unstandardized indirect effects were calculated for each of the 1,000 bootstrapped samples, and the 95% confidence interval was determined by considering the indirect effects at the 2.5th and 97.5th percentiles. The bootstrapped unstandardized indirect effect was − 2.39, with the 95% confidence interval ranging from − 5.06 to -0.95. Thus, the indirect effect was statistically significant (*p <* .001) and mediated 44% of the total effect.

A VIF value of *1.09* showed that collinearity was not a problem for our model.

### Depression

**Fig. 3:**
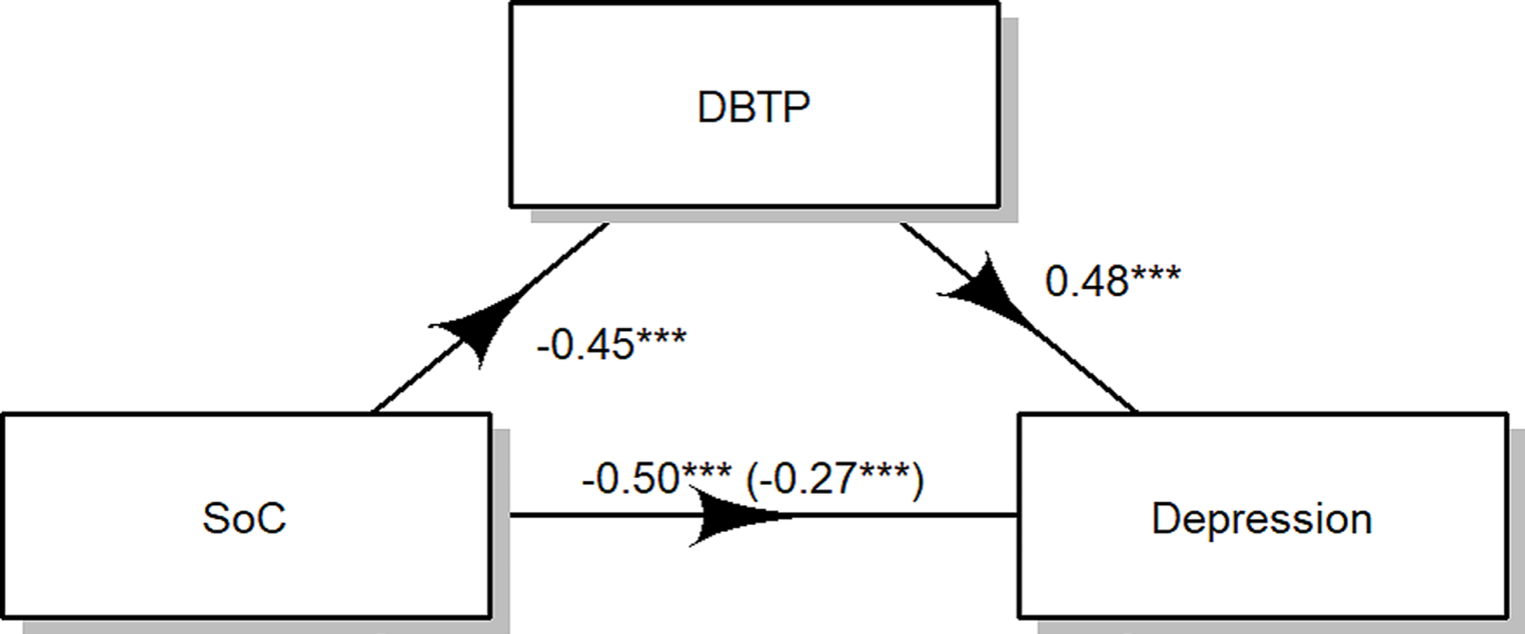
Mediation Model of DBTP on the effect of SoC on depression. *Note* *** indicates p values < 0.001

The results indicate that the influence of SoC on depression is mediated by DBTP. As illustrated in Fig. 3, both the regression coefficient between SoC and depression (*B*=-0.27, β=-0.49, t(351)=-2.97, D = 57.80, R^2^ = 0.01, *p <* .01) and the regression coefficient between DBTP and depression (*B* = 0.48, β = 0.86, t(352) = 8.29, D = 57.80, R^2^ = 0.01, p *<* .001) were found to be statistically significant. The estimated indirect effect was − 7.70. To assess the significance of this effect, bootstrapping procedures were employed. Unstandardized indirect effects were computed for each of the 1,000 bootstrapped samples, and the 95% confidence interval was established by considering the indirect effects at the 2.5th and 97.5th percentiles. The bootstrapped unstandardized indirect effect was − 7.70, with the 95% confidence interval ranging from − 14.95 to -3.52. Thus, the indirect effect was deemed statistically significant (p *<* .001) and mediated the 44% of the total effect.

A VIF value of *1.09* showed that collinearity was not a problem for our model.

### Stress

**Fig. 4:**
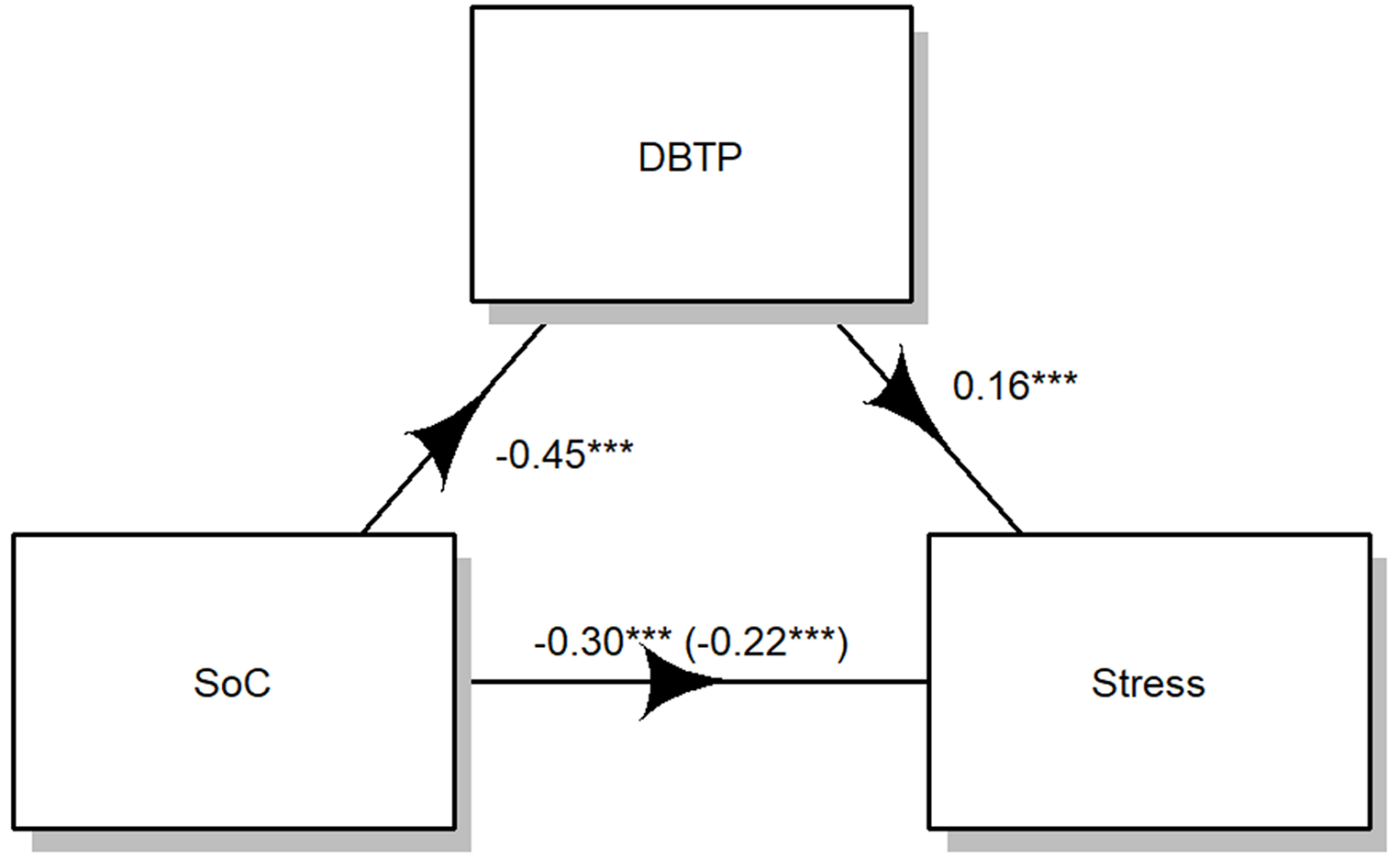
Mediation Model of DBTP on the effect of SoC on stress. *Note* *** indicates p values < 0.001

The results indicate that the influence of SoC on stress is mediated by DBTP. As illustrated in Fig. 4, both the regression coefficient between SoC and stress (*B*=-0.22, β=-0.77, t(351)=-3.35, D = 10.87, R^2^ = 0.02, *p <* .001) and the regression coefficient between DBTP and stress (*B* = 0.16, β = 0.58, t(351) = 3.98, D = 10.87, R^2^ = 0.02, p *<* .001) were found to be statistically significant. The estimated indirect effect was − 2.40. To assess the significance of this indirect effect, bootstrapping procedures were employed. Unstandardized indirect effects were computed for each of the 1,000 bootstrapped samples, and the 95% confidence interval was established by considering the indirect effects at the 2.5th and 97.5th percentiles. The bootstrapped unstandardized indirect effect was − 2.40, with the 95% confidence interval ranging from − 4.11 to -1.04. Thus, the indirect effect was deemed statistically significant (p *<* .001) and mediated the 25% of the total effect.

A VIF value of *1.09* showed that collinearity was not a problem for our model.

## Discussion

The present study aims at investigating the role of the SOC on anxiety, depression, and stress and to test whether this effect is mediated by a more balanced time perspective. Previous findings have highlighted the relationship between a balanced time perspective and anxiety, depression, and stress [31], as well as the effect of SOC on depression [17]. To date, the relationship between sense of community, anxiety, and stress has been explored by a small number of studies, mainly based on specific samples [18–21]. The results of these studies, which show the negative relationship between SOC and depression, anxiety, and distress, highlight the necessity to study these relationships in community samples, to increase the extension of the results to the general population. The mediation effect of DBTP on the effect that sense of community has on anxiety, depression, and stress has not been tested yet.

In our study, participants who reported a higher SOC, therefore experiencing a deeper feeling of belongingness to their social context, a stronger bond with community members, and a stronger commitment and belief that group members have towards each other, also reported lower levels of depression, stress, and anxiety. In this way, our results extend this previous knowledge not only by confirming the link between SOC and depression, and stress but also by testing it on a community sample, thus covering a gap in the existing literature. Indeed, previous findings have shown that the sense of community is a good predictor of the level of depression. Fowler and colleagues [17] found that the more individuals felt to be part of their community, the lower their levels of depression.

Further, previous findings also highlighted the role that a more balanced time perspective has in predicting psychological well-being [7, 9]. More in detail, it was observed that a more balanced time perspective was the best predictor of anxiety, depression, and stress in different works (see Stolarski et al. [31]). Our results, in line with this literature, showed that a higher deviation of the time perspective from the optimal levels was associated with higher levels of anxiety, depression, and stress.

However, more germane to this work were the results from the mediation models. With respect to Zimbardo’s conceptualization of Time Perspective and in accordance with previous findings, we aimed to test a possible mediation role of DBTP on the effect that the SOC has on anxiety, depression, and stress. In their conceptualization of the five time perspectives, indeed, Zimbardo & Boyd [7] assumed that different psychological domains can concur on the development of a specific time perspective. In the specific, time perspective arises from the combination of motivational, emotional, cognitive, and social processes that contribute and, in turn, are influenced by, its development. Moreover, time perspective is “*an integral part of a society’s values and individuals orient their action in the present and toward the future with reference to the groups whose values they share*” [32, p.192]. In this way, it is worthy to understand whether sense of community predicts the deviation from a balanced time perspective. In addition, it is also relevant to see whether this effect contributes to explaining the effects that sense of community has on psychological well-being. Based on previous work on the mediation effects of time perspective, we hypothesize that there is, indeed, a full mediation effect of the deviation from a balanced time perspective on the levels that the sense of community has on anxiety, depression, and stress. It has been found, indeed, a significant mediation effect of time perspective on the effects that childhood traumas [33] and quality of life [34] have on depressive symptomatology, showing, for instance, that an abnormal time perspective, oriented more towards the past and less towards the future, was a mediator of the depressive symptomatology [33]; as well as a more negative time perspective mediated the effect that quality of life has on depressive symptomatology in cancer survivors [34].

Interestingly, our results showed that the relationship between sense of community and psychological distress was mediated by the deviation from a balanced time perspective extending previous findings and enriching the existing literature on time perspective. Taken together, our findings not only show the importance that a stronger sense of community has on psychological well-being but also that this effect is fully mediated by the deviation from a balanced time perspective.

Overall, this study has some limitations. First, the data were collected using self-report measures, which may suffer from memory and response bias, at least to a certain degree [35]. Second, the cross-sectional design did not allow to infer causality, thus longitudinal research is needed to confirm the directionality of the hypothesized associations. Third, the study e was conducted on an Italian Community sample, therefore for a better generalization of the results further research should be conducted in also different countries. A final limitation concerns the sample size employed in the present study. Here, we used a convenience sample of 352 subjects obtained in a wider data collection started during COVID-19 restrictions. Here we only considered the participants who completed the survey from July 2020 to October 2020, outside of the Italian restrictions period. In this way, we reduced the number of observations, although we excluded possible confounders due to the restrictions [36].

Concluding, the results described so far could be applied to build a series of interventions aimed at promoting psychological well-being in the general population. Due to the mediation effects that a more balanced time perspective has on the relationship between sense of community and psychological well-being, it would be beneficial, for instance, to develop training programs aimed at prompting and informing individuals on time perspective. For instance, from a more therapeutic point of view, a work based on time perspective restructuring has been proven to be effective on health conditions such as post-traumatic stress disorder, improving the individual’s psychological well-being and decreasing the characteristic symptomatology [37]. Considering our findings, we suggest that individuals’ health could be promoted by both improving their sense of community, which in turn would decrease their levels of stress, and by restructuring their time perspective when it became dysfunctional and unbalanced so that this approach individuals adopt in considering their life experiences can serve as a coping strategy to face more complex situations.

## Data Availability

You can find Data related to the present work at https://osf.io/2qbaw/?view_only=8984b1a2f7544a7d9dde7652c941a5ca.

## Abbreviations

ZTPI: Zimbardo Time Perspective Inventory
BTP: Balanced Time Perspective
DBTP: Deviation from a Balanced Time Perspective
PN: Past Negative
PP: Past Positive
F: Future
PH: Present Hedonistic
PF: Present Fatalistic
SOC: Sense of Community
DASS: 21–Depression Anxiety and Stress Scale 21 item
MTSOCS: Multidimensional Sense of Community Scale
VIF: Variance Inflation Factor
D: Deviance

## References

[1] World Health Organization (2017). Depression and other Common Mental disorders: Global Health estimates.

[2] World Health Organization. COVID-19 pandemic triggers 25% increase in prevalence of anxiety and depression worldwide. Retrieved Dec 21, 2023. http://tinyurl.com/mw859ssn.

[3] Kessler RC, Aguilar-Gaxiola S, Alonso J, Benjet C, Bromet EJ, Cardoso G (2017). Trauma and PTSD in the WHO world mental health surveys. Eur J Psychotraumatology.

[4] Lépine Jean-Pierre (2002). The epidemiology of anxiety disorders: prevalence and societal costs. Journal of Clinical Psychiatry.

[5] Bandelow B, Michaelis S (2015). Epidemiology of anxiety disorders in the 21st century. Dialog Clin Neurosci.

[6] American Psychiatric Association. Diagnostic and statistical Manual of Mental disorders. 5th ed. American Psychiatric Association; 2022. Text Revision.

[7] Zimbardo PG, Boyd JN (1999). Putting time in perspective: a Valid, Reliable Individual-differences Metric. J Personal Soc Psychol.

[8] McMillan DW, Chavis DM (1986). Sense of community: a definition and theory. J Community Psychol.

[9] Boniwell I, Zimbardo P. Time to find the right balance. The psychologist. 2003.

[10] Boniwell I, Zimbardo PG. Balancing time perspective in pursuit of optimal functioning. Positive psychology in practice: Promoting human flourishing in work, health, education, and everyday life. 2015;pp. 223–236.

[11] Cacioppo JT, Patrick W. Loneliness: human nature and the need for social connection. WW Norton & Company; 2008.

[12] Desmyter F, De Raedt R (2012). The relationship between time perspective and subjective well-being of older adults. Psychologica Belgica.

[13] Papastamatelou J, Unger A, Giotakos O, Athanasiadou F (2015). Is time perspective a predictor of anxiety and perceived stress? Some preliminary results from Greece. Psychological Studies.

[14] Micillo L, Rioux PA, Mendoza E, Ku¨bel SL, Cellini N, Van Wassenhove V (2022). Time perspective predicts levels of anxiety and depression during the COVID-19 outbreak: a cross-cultural study. PLoS ONE.

[15] Peterson NA, Speer PW, McMillan DW (2008). Validation of a brief sense of community scale: confirmation of the principal theory of sense of community. Journal of Community Psychology.

[16] Greenfield EA, Marks NF (2010). Sense of community as a protective factor against long-term psychological effects of childhood violence. Social Service Review.

[17] Fowler K, Wareham-Fowler S, Barnes C (2013). Social context and depression severity and duration in Canadian men and women: exploring the influence of social support and sense of community belongingness. Journal of Applied Social Psychology.

[18] Li Y, Sun F, He X, Chan KS (2011). Sense of community and depressive symptoms among older earthquake survivors following the 2008 earthquake in Chengdu China. Journal of Community Psychology.

[19] García-Cid A, Gómez-Jacinto L, Hombrados-Mendieta I, Millán-Franco M, Moscato G (2020). Discrimination and psychosocial well-being of migrants in Spain: the moderating role of sense of community. Frontiers in Psychology.

[20] Benson OM, Whitson ML (2022). The protective role of sense of community and access to resources on college student stress and COVID-19-related daily life disruptions. Journal of Community Psychology.

[21] Witting AB, Busby DM, Allen E. Sense of community and anxiety during a global pandemic: the role of world assumptions in couples. Stress Health. 2023. 10.1002/smi.3235 36790741

[22] Lovibond SH. Manual for the depression anxiety stress scales. Sydney psychology foundation. 1995.

[23] Bottesi G, Ghisi M, Altoé G, Conforti E, Melli G, Sica C (2015). The Italian version of the Depression anxiety stress Scales-21: factor structure and psychometric properties on community and clinical samples. Comprehensive Psychiatry.

[24] Mioni G, Wittmann M, Prunetti E, Stablum F (2020). Time perspective and the subjective passage of time in patients with borderline personality disorders. Timing &amp; Time Perception.

[25] Prezza M, Pacilli MG, Barbaranelli C, Zampatti E (2009). The MTSOCS: a multidimensional sense of community scale for local communities. Journal of Community Psychology.

[26] Jankowski KS, Zajenkowski M, Stolarski M (2020). What are the optimal levels of time perspectives? Deviation from the balanced time perspective-revisited (DBTP-r). Psychologica Belgica.

[27] R Core Team. R: A Language and Environment for Statistical Computing. Vienna, Austria. https://www.R-project.org/.

[28] Tingley D, Yamamoto T, Hirose K, Keele L, Imai K, Mediation. R package for causal mediation analysis. 2014;R package version 4.4.7.

[29] Fox J, Weisberg S, Adler D, Bates D, Baud-Bovy G, Ellison S et al. Package ’car’. Vienna. Version 2.1-6. https://cran.r-project.org/web/packages/car/index.html.

[30] Craney TA, Surles JG (2002). Model-dependent variance inflation factor cutoff values. Qual Eng.

[31] Stolarski M, Zajenkowski M, Jankowski KS, Szymaniak K (2020). Deviation from the balanced time perspective: a systematic review of empirical relationships with psychological variables. Personality and Individual Differences.

[32] Coser L, Coser R. Time perspective and social structure. The sociology of time. Springer; 1990. pp. 191–202.

[33] Wang Y, Hu X, Han J, Scalabrini A, Hu Y, Hu Z (2021). Time is of essence-abnormal time perspectives mediate the impact of childhood trauma on depression severity. Journal of Psychiatry Research.

[34] Bitsko MJ, Stern M, Dillon R, Russell EC, Laver J (2008). Happiness and time perspective as potential mediators of quality of life and depression in adolescent cancer. Pediatr Blood Cancer.

[35] Chan D, Lance CE, Vandenberg RJ (2009). So why ask me? Are self report data really that bad?. Statistical and methodological myths and urban legends: Doctrine, verity and fable in the organizational and social sciences.

[36] Lakens D (2022). Sample size justification. Collabra Psychology.

[37] Zimbardo P, Sword R, Sword R. The time cure: overcoming PTSD with the new psychology of time perspective therapy. Wiley; 2012.

